# Metabolic reprogramming as a novel therapeutic target for Coxsackievirus B3

**DOI:** 10.1080/19768354.2022.2141318

**Published:** 2022-11-03

**Authors:** Myeong Uk Kuk, Yun Ji Ga, Ye Jin Kim, Ji Yun Park, Eun Seon Song, Haneur Lee, Yun Haeng Lee, Gahyun Ko, Jae Kwang Kim, Jung-Yong Yeh, Hyung Wook Kwon, Youngjoo Byun, Joon Tae Park

**Affiliations:** a Division of Life Sciences, College of Life Sciences and Bioengineering, Incheon National University, Incheon, Korea; b Convergence Research Center for Insect Vectors, Incheon National University, Incheon, Korea; c College of Pharmacy, Korea University, Sejong, Republic of Korea

**Keywords:** Coxsackievirus B3, metabolic alteration, glycolysis inhibitors, antiviral effect

## Abstract

Coxsackievirus B3 (CVB3) is a single-stranded RNA virus that belongs to the *Enterovirus* genus. CVB3 is a human pathogen associated with serious conditions such as myocarditis, dilated cardiomyopathy, and pancreatitis. However, there are no therapeutic interventions to treat CVB3 infections. In this study, we found that CVB3 induced metabolic alteration in host cells through increasing glycolysis level, as indicated by an increase in the extracellular acidification rate (ECAR). CVB3-mediated metabolic alteration was confirmed by metabolite change analysis using gas chromatography-mass spectrometry (GC-MS). Based on findings, a strategy to inhibit glycolysis has been proposed to treat CVB3 infection. Indeed, glycolysis inhibitors (2-Deoxy-D-glucose, sodium oxide) significantly reduced CVB3 titers after CVB3 infection, indicating that glycolysis inhibitors can be used as effective antiviral agents. Taken together, our results reveal a novel mechanism by which CVB3 infection is controlled by regulation of host cell metabolism.

## Introduction

Viruses are intracellular parasites that depend on the physiology of infecting host cells for survival (Lozach 2020). Their replication capacity is strongly influenced by host cell responses such as availability of macromolecules and innate antiviral defenses (Barr and Fearns 2016). In addition to being affected by host cell responses, viruses affect host cell responses by modifying cell metabolism, which mainly consists of oxidative phosphorylation (OXPHOS) and glycolysis (Cassim et al. 2018). For example, human cytomegalovirus infection exhibited higher glucose dependence and upregulated glycolysis in host cells (Munger et al. 2006; Ripoli et al. 2010). Similarly, viruses such as herpes simplex virus 1, rubella virus, dengue virus, rhinovirus, influenza virus, vitiligo virus, and adenovirus induced host cell responses characterized by increased glycolysis (Abrantes et al. 2012; Barrero et al. 2013; Passalacqua et al. 2019). This phenomenon is intended to compensate for the energy deficit caused by virus-mediated mitochondrial dysfunction. Specifically, viruses interact with components of the electron transport complex and inner mitochondrial membrane complexes, rapidly lowering OXPHOS efficiency (Cavallari et al. 2018). The deterioration in OXPHOS efficiency reprograms the host cell to increase its dependence on glycolysis as an energy source (Moreno-Altamirano et al. 2019). Therefore, unraveling the specific metabolic responses that regulate OXPHOS and glycolysis may help develop broad-acting antiviral therapeutics.

Coxsackievirus B3 (CVB3) is a single-stranded RNA virus that belongs to the *Enterovirus* genus (Garmaroudi et al. 2015). CVB3 causes a variety of human disorders, ranging from the minor (rash and myalgia) to the lethal (aseptic meningitis, chronic myocarditis, etc.) (Garmaroudi et al. 2015). Evolution has enabled CVB3 to employ a variety of strategies, including exacerbating target organ tissues and inducing apoptosis in host cells. Several studies have reported several pharmacological options for blocking CVB replication *in vitro*, targeting various stages of the CVB3 life cycle (Garmaroudi et al. 2015). However, no drug has received end-market approval due to side effects or unsatisfactory antiviral activity (Garmaroudi et al. 2015). Given these findings, there is a need for a more effective candidates that can be used for CVB3 treatment.

In this study, we aimed to elucidate the host cell responses that CVB3 induces to promote disease. Here, we identified the CVB3-mediated metabolic alteration to glycolysis and propose the use of glycolysis inhibitor as an effective treatment for CVB3. Our results will provide a novel strategy to control CVB3 infection through regulation of host cell metabolism.

## Materials and methods

### Cell culture and viral infection

Hela cells (CCL-2™; ATCC, Manassas, Virginia, USA) were cultured in Dulbecco’s modified Eagle’s medium (DMEM; 11995065, Invitrogen Life Technologies, Carlsbad, CA, USA) supplemented with 10% fetal bovine serum (16000036; Invitrogen Life Technologies) and a 100 μg/ml penicillin/streptomycin cocktail (15240062; Invitrogen Life Technologies) in an atmosphere comprising 5% CO_2_. Hela cells were infected with Coxsackievirus B3 (CVB3; 41202, National Culture Collection for Pathogens, Osong-eup, Korea) at a multiplicity of infection (MOI) of 0.1 for 1 h. As a mock control, Hela cells were treated with phosphate buffered saline (PBS; 14190144; Invitrogen Life Technologies) for 1 h. To investigate the effect of glucose on CVB3 infection, Hela cells were treated with glucose-free DMEM (11966025, Invitrogen Life Technologies) after adding different concentrations of glucose (A2494001; Invitrogen Life Technologies; 0, 1 g/L). To study the effect of glucose on CVB3 infection, Hela cells were incubated with glucose-free DMEM (11966025; Invitrogen Life Technologies) containing different concentrations of glucose (A2494001; Invitrogen Life Technologies; 0, 1 g/L). Moreover, after fixing the glucose concentration in the medium to 1 g/L, Hela cells were treated with 10 mM 2-deoxy-D-glucose (2-DG; D8375; Sigma–Aldrich, St. Louis, MO, USA) or 10 mM sodium oxamate (SO; O2751; Sigma–Aldrich) to study the effect of glycolysis inhibition on CVB3 infection.

### Apoptosis assay

Apoptosis assay using the FITC Annexin V Apoptosis Detection Kit (556547; BD Biosciences, Franklin Lakes, NJ, USA) was measured according to manufacturer’s instructions (Park et al. 2021).

### Measurement of mitochondrial membrane potential (MMP)

To quantify MMP, cells were treated for 30 min at 37°C with 0.3 g/ml JC-1 (T3168; Invitrogen Life Technologies) and then processed for flow cytometry analysis as described before (Park et al. 2020).

### Analysis of the extracellular acidification rate (ECAR)

The XFe24 flux analyzer and a prep station (Seahorse Bioscience XFe24 Instrument; Seahorse Bioscience, Billerica, MA, USA) were used. Briefly, 5 × 10^4^ cells were placed in each well of an XFe24 cell-culture plate (100850-001; Seahorse Bioscience) and examined as previously described (Kim et al. 2019).

### Metabolite extraction and GC-MS analysis

The hydrophilic metabolites from the cells were extracted by modifying a previously described (Kim et al. 2017). The sample was transferred into auto-sampler vials and analyzed by gas chromatography-mass spectrometry (GC-MS, a GCMS-QP2010 Ultra system, Shimadzu, Kyoto, Japan). GC-MS analysis was performed as previously described (Song et al. 2021). Pearson's correlation analysis was carried out using SAS software 9.4 (SAS Institute, San Diego, CA, USA) and then visualized with Hierarchical Clustering Analysis (HCA) using Multi-Experiment Viewer version 4.9.0 (MeV; https://webmev.tm4.org).

### Plaque assay

The virus titer in the cell supernatant was determined as previously described (Wong et al. 2008). In brief, the supernatant from CVB3-infected cells was serially diluted 10-fold before being overlaid on a Vero cell monolayer that was 90–95% confluent. The Vero cells were rinsed in PBS after 1 h incubation, then overlayed with a 2 × DMEM (LM 20150; Welgene, Gyeongsan, Korea) / 2% agar (50100; Lonza, Walkersville, MD, USA) mixture. The cells were fixed with 3.75% formaldehyde (F8775; Sigma–Aldrich) solution and stained with 1% crystal violet (V5265; Sigma–Aldrich) after being cultured at 37°C for 72 h. The virus titer was determined as plaque-forming unit (PFU) per milliliter after the plaques were counted.

### Statistical analyses

Statistical analyses were performed using a standard statistical software package (SigmaPlot 12.5; Systat Software, San Jose, CA, USA). The Student’s t-test or two-way ANOVA followed by Bonferroni’s post test was used to determine whether differences were significant.

## Results

### CVB3 induces metabolic changes through an increase in glycolysis

**Figure 1.**
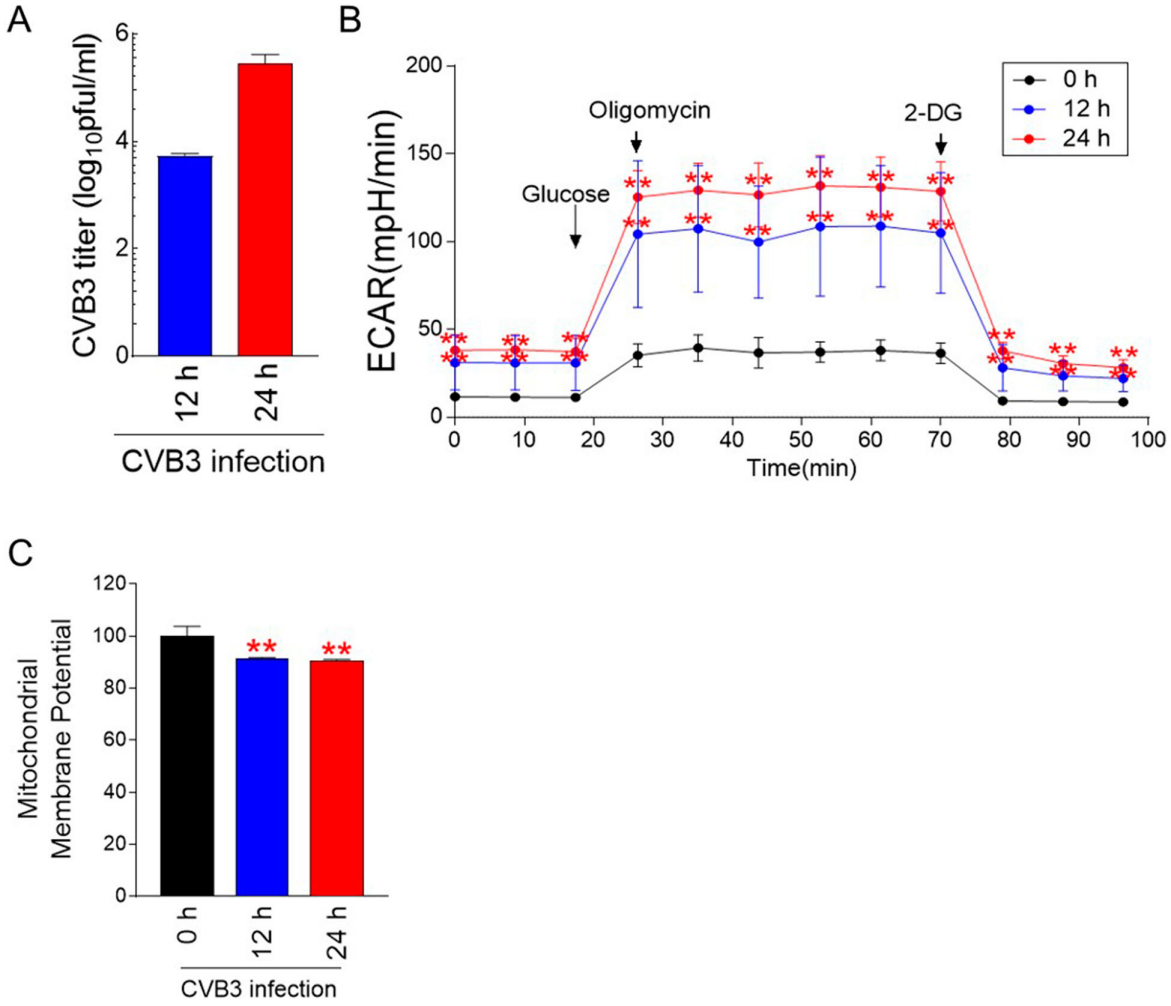
CVB3 induces metabolic alteration through an increase in glycolysis. (A) Hela cells were infected with CVB3 for 12 h or 24 h. Then, plaque assays were then performed to quantify CVB3 titers. Means ± S.D., *N* = 3. (B) Measurement of the extracellular acidification rate (ECAR) (black line: post infection 0 h, blue line: post infection 12 h, and red line: post infection 24 h) (**P < 0.01, two-way ANOVA followed by Bonferroni’s post test). Means ± S.D., *N* = 3. (C) Flow cytometric analysis of mitochondrial membrane potential using JC-1 (**P < 0.01, Student’s t-test). Means ± S.D., *N* = 3.

As metabolic reprogramming is one of the host cell responses induced by viruses (Thaker et al. 2019), we examined whether CVB3 induced metabolic alteration in host cells. Hela cells were infected with CVB3 for 12 h or 24 h. Thereafter, the extracellular virus was collected from the supernatant and tested using a plaque assay to determine the titer. CVB3 titers increased in a time-dependent manner (Figure 1A).

To monitor changes in energy metabolism, glycolysis levels including glycolysis, glycolytic capacity and glycolytic reserve were assessed by measuring the extracellular acidification rate (ECAR) (Brand and Nicholls 2011). Glycolysis was assessed by injecting glucose that cells metabolize for glycolysis (Brand and Nicholls 2011). Then, oligomycin, which inhibits mitochondrial ATP production, was injected to evaluate glycolytic capacity. Finally, glycolytic reserve was evaluated by injecting 2-deoxy-glucose (2-DG), which inhibits glycolysis through competitively binding to hexokinase II. CVB3 infection significantly increased ECAR values at both 12 and 24 h p.i. (Figure 1B). These data indicate that CVB3 infection induced metabolic changes through an increase in glycolysis levels including glycolysis, glycolytic capacity and glycolytic reserve.

Mitochondrial membrane potential (MMP) is electrochemical gradient across the inner mitochondrial membrane, leading to ATP synthesis during OXPHOS (Gupta et al. 2009). To indirectly measure OXPHOS efficiency, we examined MMP after CVB3 infection. CVB3 significantly decreased MMP in host cells at both 12 and 24 h p.i. (Figure 1C).

### Confirmation of glycolysis induction by CVB3 through a metabolic analysis

**Figure 2.**
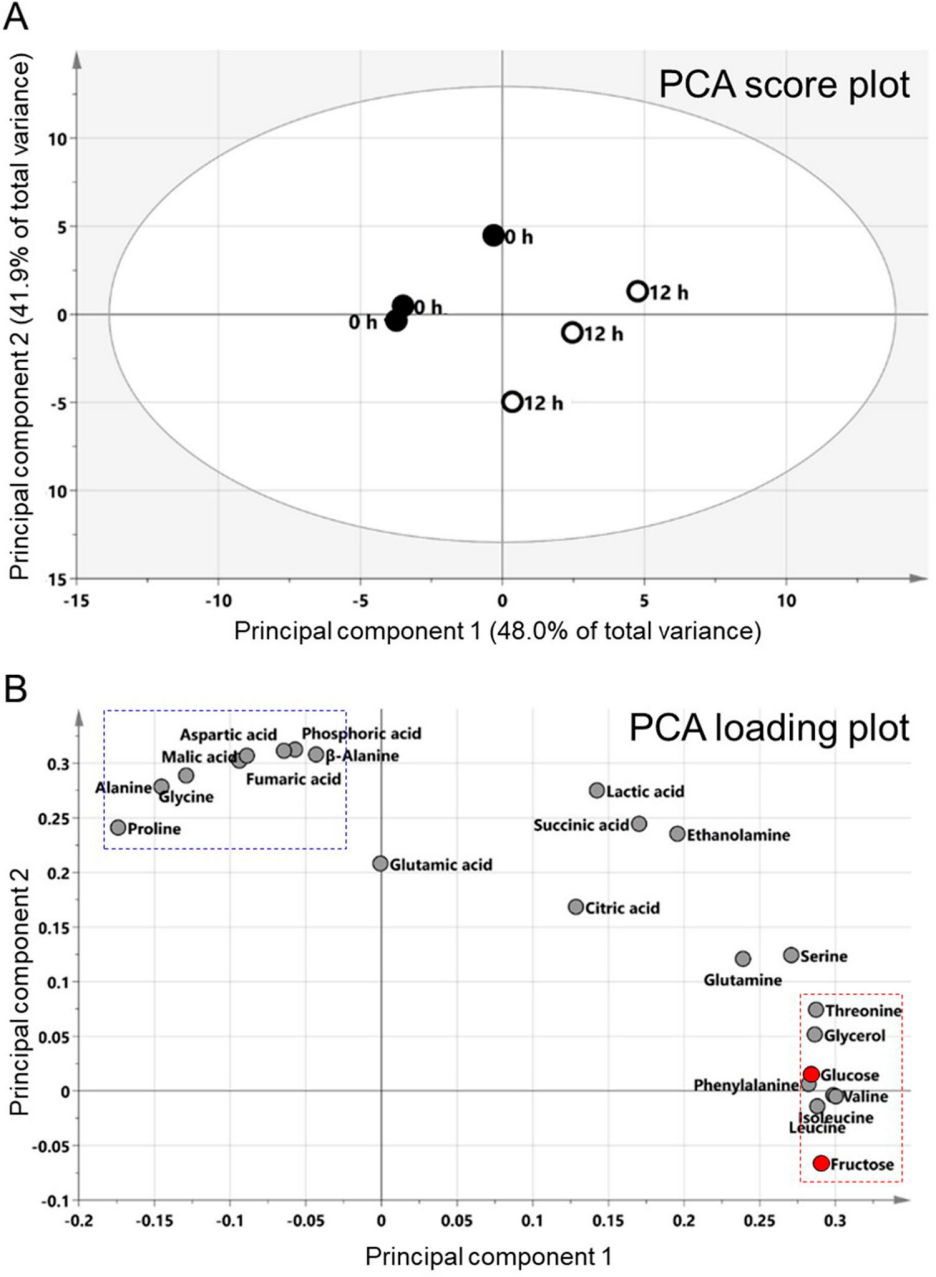
Confirmation of CVB3-mediated metabolic changes through metabolite analysis. Principal component analysis (PCA) score plots (A) and loading plots (B) derived from 23 metabolites. When metabolites belonging to PC1 were classified according to similarity, fructose, leucine, isoleucine, valine, phenylalanine, glucose, glycerol and threonine were classified as one cluster (red rectangle). Moreover, regarding to PC2, aspartic acid, malic acid, phosphoric acid, fumaric acid, β-alanine, alanine, proline and glycine were classified one cluster (blue rectangle).

To confirm CVB3-mediated metabolic alteration, we analyzed metabolite changes using gas chromatography-mass spectrometry (GC-MS). Since significant changes in glycolysis levels were observed from 12 h p.i., we hypothesized that focusing on the difference in metabolites between 0 and 12 h p.i. would yield meaningful outcomes. Thus, metabolites were analyzed at 0 and 12 h time points. Pearson's correlation analysis (PCA) was performed to visualize potential metabolite differences between the two groups. PCA revealed that the two highest-ranking principal components (PC) accounted for 89.9% of the total variance on the PCA score plot (PC1, 48.0%; PC2, 41.9%) (Figure 2A). Both PC1 and PC2 separated the 0 and 12 h p.i. groups, indicating differences in metabolites between the two groups (Figure 2A). Furthermore, the PCA loading plot indicated the major factors associated with the differences were 23 metabolites, including 13 amino acids, 6 organic acids, 2 sugars, 1 amine, and 1 sugar alcohol (Figure 2B). When metabolites belonging to PC1 were classified according to similarity, fructose, leucine, isoleucine, valine, phenylalanine, glucose, glycerol and threonine were classified as one cluster (Figure 2B; red rectangle). This cluster contained glucose, a main fuel source for glycolysis and fructose, a major source of fructose-6-phosphate, an intermediate for glycolysis (Figure 2B; red dot). Moreover, when metabolites belonging to PC2 were classified according to similarity, aspartic acid, malic acid, phosphoric acid, fumaric acid, β-alanine, alanine, proline and glycine were classified one cluster (Figure 2B; blue rectangle).

**Figure 3.**
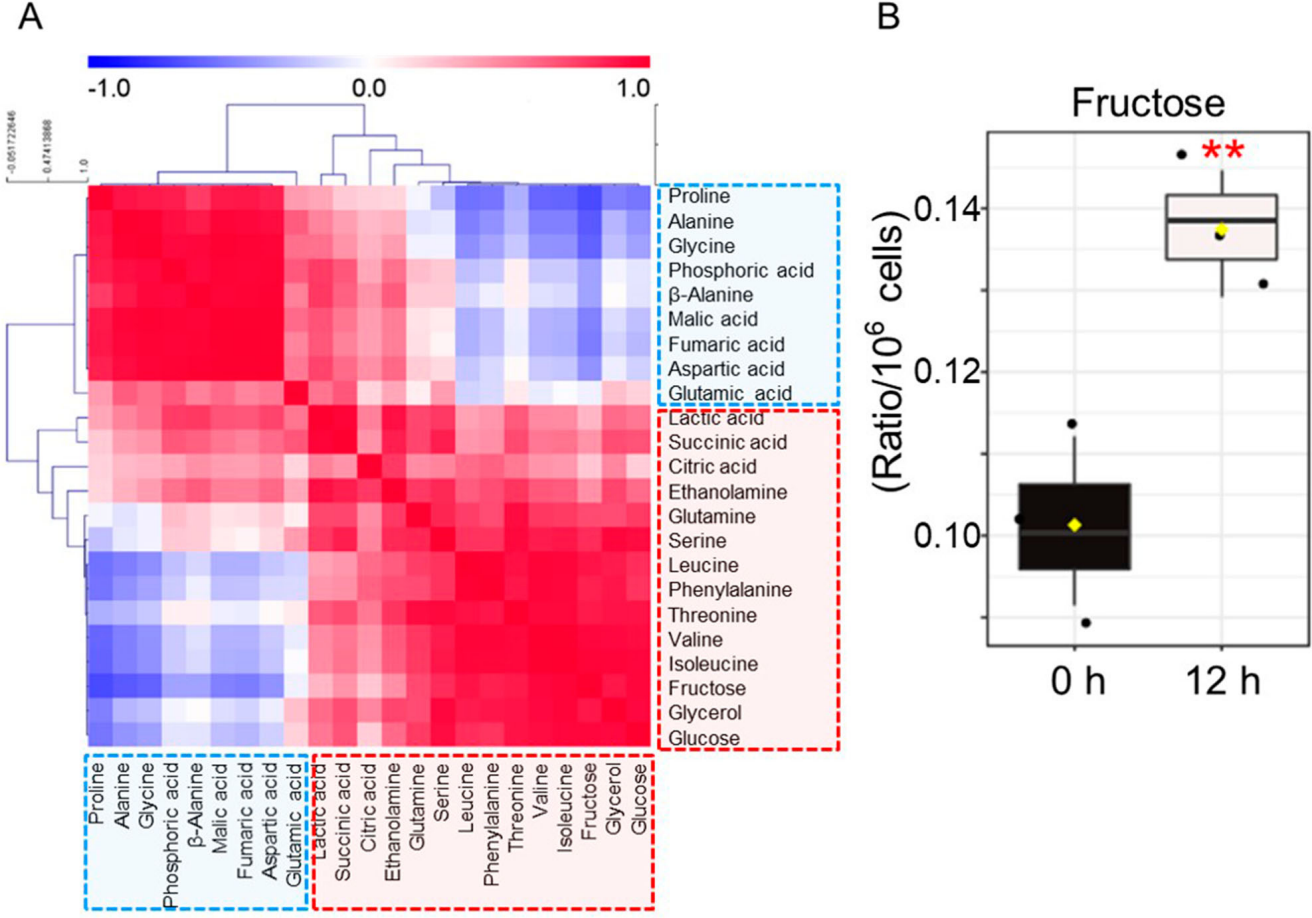
Confirmation of CVB3-mediated metabolic changes through metabolite analysis. (A) Correlation matrix of 23 metabolites. The intensity of the blue or red color in each square represents the Pearson's correlation coefficient. One group, shown mostly in blue, comprised 6 amino acids (proline, alanine, glycine, β-alanine, aspartic acid, glutamic acid) and 3 organic acids (phosphoric acid, malic acid, fumaric acid). The other group, shown mostly in red, contained 7 amino acids (glutamine, serine, leucine, phenylalanine, threonine, valine, isoleucine), 3 organic acids (lactic acid, succinic acid, citric acid), 2 sugars (fructose, glucose), 1 amine (ethanolamine) and 1 sugar alcohol (glycerol). (B) Box plot of metabolite with the most significant change after CVB3 infection (**P < 0.01, Student’s *t*-test). Means ± S.D., *N* = 3.

PCA results were assigned to Hierarchical Cluster Analysis (HCA) and displayed in color, with red for positive correlation and blue for negative correlation. One group, shown mostly in blue, comprised 6 amino acids (proline, alanine, glycine, β-alanine, aspartic acid, glutamic acid) and 3 organic acids (phosphoric acid, malic acid, fumaric acid) (Figure 3A). The other group, shown mostly in red, contained 7 amino acids (glutamine, serine, leucine, phenylalanine, threonine, valine, isoleucine), 3 organic acids (lactic acid, succinic acid, citric acid), 2 sugars (fructose, glucose), 1 amine (ethanolamine) and 1 sugar alcohol (glycerol) (Figure 3A). Among the groups shown in red, 2 sugars (fructose, glucose) were the most positively correlated (Figure 3A; *r* = 0.9425, *P* < 0.01). Among two components, fructose, a major source of fructose-6-phosphate, was the metabolite with the most significant change after CVB3 infection (Figure 3B; *P* < 0.01). These data confirmed that CVB3 induced metabolic alterations through an increase in glycolysis dependence.

### Glycolysis inhibitors as novel therapeutic strategies for CVB3 treatment

**Figure 4.**
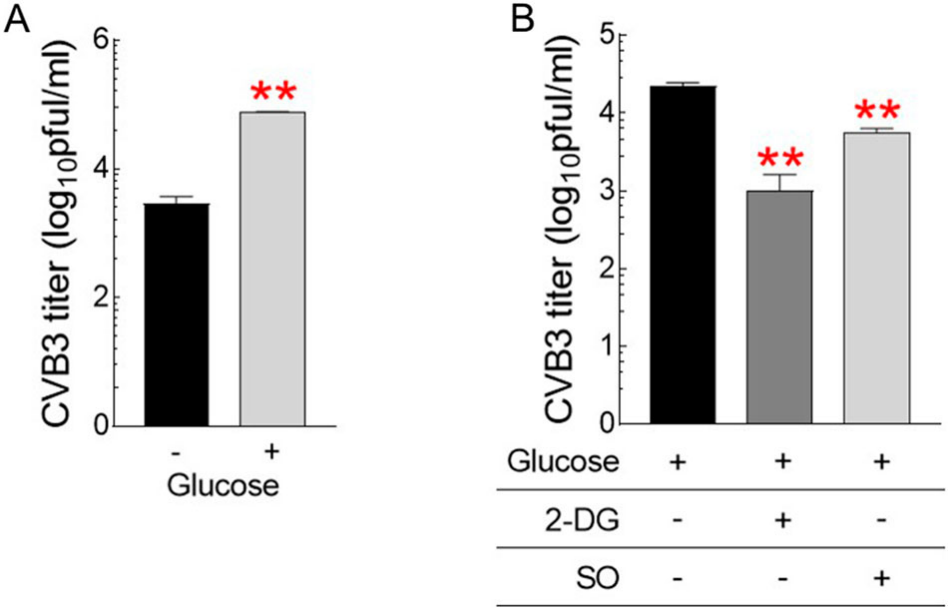
Glycolysis inhibitors as novel therapeutic strategies for CVB3 treatment. (A) Quantification of CVB3 titers in Hela cells treated with medium with or without glucose (1 g/L) (**P < 0.01, Student’s t-test). Means ± S.D., N = 3. (B) Quantification of CVB3 titers in Hela cells treated with glycolysis inhibitors, 2-deoxy-D-glucose (2-DG) or sodium oxamate (SO) after fixing the glucose concentration in the medium to 1 g/L. (**P < 0.01, Student’s t-test). Means ± S.D., *N* = 3.

Identification of CVB3-mediated metabolic alteration to glycolysis led to the hypothesis that strategies to reduce glycolysis dependence would be useful for CVB3 therapy. To prove the concept of our hypothesis, we investigated the effect of glucose on CVB3 infection by treating cells with different glucose concentrations or cells with glycolysis inhibitors. First, cells were treated with medium with or without glucose (1 g/L). Then, CVB3 titers were investigated 72 h after CVB3 infection. CVB3 titers were 10-fold higher in the presence of glucose than in the absence, indicating that glucose provides an environment for CVB3 to actively replicate in the host cell (Figure 4A). Second, after fixing the glucose concentration in the medium to 1 g/L, cells were further treated with well-characterized glycolysis inhibitors, 2-DG or sodium oxamate (SO) (Zhang et al. 2014; Qiao et al. 2021). Inhibition of glycolysis by 2-DG or SO significantly reduced CVB3 titers compared to controls, suggesting that strategies utilizing glycolysis inhibitors would be beneficial in therapeutic approaches for CVB3 treatment (Figure 4B).

## Discussion

Metabolic reprogramming is one of the host cell responses induced by viruses to intercept host cell machinery (Thaker et al. 2019). Viruses rapidly compensate for the lack of energy for survival by altering host cell metabolism, which consists primarily of OXPHOS and glycolysis (Weckmann et al. 2018). CVB3 also evolves molecular mechanisms that utilize the various compartments of the host cell (Garmaroudi et al. 2015). However, it is not well known whether CVB3 induces metabolic reprogramming in host cells during viral replication and proliferation. In the present study, we discovered that CVB3 induced metabolic alteration through increasing glycolysis levels. CVB3-mediated increase in glycolysis was assessed by measuring glycolysis level based on ECAR value and analyzing metabolites. Moreover, the deterioration of OXPHOS was assessed indirectly by identifying the CVB3-mediated reduction of MMPs, which resulted in a lower electrochemical gradient across the inner mitochondrial membrane. To our knowledge, this is the first work to show that CVB3 upregulates glycolysis in host cells to compensate for the lack of energy sources caused by CVB3 infection. A deeper understanding of how CVB3 alters metabolic pathways might lead to more potent CVB3 antiviral treatment.

Host cell metabolism is considered a target of viral infection, and appropriate modulation of it has been proposed to provide an effective treatment against the virus. The close relationship between host cell metabolism and viral therapy was demonstrated by the discovery that the metabolic regulation of host cells was an intrinsic factor controlling murine norovirus (MNV) replication (Passalacqua et al. 2019). Thus, as a novel therapeutic target for MNV infection, glycolysis has been proposed. Indeed, inhibition of glycolysis by 2-DG affected the MNV uptake and capsid coating removal process (Passalacqua et al. 2019). The significance of host cell metabolism as a viral therapy is further supported by the finding that glycolysis flux is essential for survival and spread of severe acute respiratory syndrome coronavirus 2 (SARS-CoV-2) (Codo et al. 2020). Inhibition of glycolysis by 2-DG abrogates SARS-CoV-2 replication, positioning glycolysis as a potential therapeutic target for SARS-CoV-2 pathogenesis (Codo et al. 2020). Herein, we provided the first demonstration that the use of glycolysis inhibitors, 2-DG or SO, significantly reduced CVB3 titers. The discovery of glycolytic inhibition-mediated reduction of CVB3 titers led to the question of whether opposite conditions could lead to opposite outcomes. Indeed, activation of glycolysis by supplementing with higher glucose concentrations increased CVB3 titers. Taken together, these results suggest that modulation of metabolic reprogramming could be effective in therapeutic approaches for CVB3 treatment.

Glycolysis inhibitors have different mechanisms of action with respect to inhibiting glycolysis. 2-DG is a glucose analog and prohibits the first rate-limiting enzyme of glycolysis, hexokinase II, which catalyzes the initial metabolic step in conversion of glucose (Yamasaki et al. 2011; Xiao et al. 2013). SO is a structural analog of pyruvate and inhibits a lactate dehydrogenase, which catalyzes the last metabolic step in conversion of pyruvate in anaerobic glycolysis (Cassim et al. 2018). In our study, we used two glycolysis inhibitors and observed different levels of reduction in CVB3 titers. Regarding the degree of change, 2-DG had a much better effect on reducing CVB3 titer than SO. Based on these data, 2-DG could be suggested as a more effective anti-CVB3 agent than SO, but should be very cautious in claims. 2-DG has poor pharmacokinetic properties, such as short half-life, making it a relatively inferior as a therapeutic agent (Hansen et al. 1984). Moreover, in order to compete with blood glucose, 2-DG should be administered at a high concentration (Strandberg et al. 2013). Furthermore, 2-DG exhibits side effects such as death of influenza virus-infected mice through promoting survival of virus-infected cells (Wang et al. 2016). These poor pharmacokinetic properties and side effects limit the clinical use of 2-DG in viral infections. As an alternative strategy, SO has been used and has shown effective results in controlling viral replication and propagation (Zhang et al. 2019; Icard et al. 2021; Zhou et al. 2021). For example, SO inhibited hepatitis B virus infection without being limited to one cell type (Zhou et al. 2021). Moreover, SO suppressed the replication of Vesicular stomatitis virus and Herpes simplex Virus-1 through decreasing lactate level (Zhang et al. 2019). Furthermore, there are no reports yet that SO can cause side effects while controlling viral infection. Given the low side effects of SO, we propose that the use of SO could be an effective treatment for CVB3 therapy.

In summary, we found that CVB3 regulate energy metabolism in host cells. The discovery of altered metabolism has led to testing the modulation of metabolism as a therapeutic option for CVB3. Indeed, the fine-tuning of metabolism by glycolysis inhibitors was effective in reducing CVB3 titers. Therefore, our results suggest that metabolic regulation through glycolysis inhibitors will serve as novel therapeutic strategies for CVB3 treatment.
